# Mediastinal bronchial artery aneurysm complicated with iatrogenic acute aortic dissection during bronchial artery embolization repaired with thoracic endovascular aortic repair: a case report

**DOI:** 10.1186/s44215-023-00125-3

**Published:** 2023-11-15

**Authors:** Hanae Higa, Takeaki Miyata, Toshifumi Saga, Takashi Yoshimatsu

**Affiliations:** 1 Department of General Thoracic Surgery, Shinkuki General Hospital, 418-1 Kamihayami, Kuki, Saitama, 346-8530 Japan; 2 Department of Cardiovascular Surgery, Shinkuki General Hospital, 418-1 Kamihayami, Kuki, Saitama, 346-8530 Japan; 3 Department of General Thoracic Surgery, Fukuoka Wajiro Hospital, 2-2-75 Wajirogaoka, Higashi-ku, Fukuoka, Fukuoka 811-0213 Japan

**Keywords:** Mediastinal bronchial artery aneurysm, BAA, Bronchial artery embolization, BAE, Thoracic endovascular aortic repair, TEVAR, Aortic stent graft

## Abstract

**Background:**

Asymptomatic bronchial artery aneurysms (BAA) could be underdiagnosed, and the precise prevalence is unknown. Bronchial artery angiography revealed that this rare disease in about 1% of patients. BAA is fatal if ruptured since there is no correlation between the diameter and symptoms of the aneurysm and the risk of rupture. Early treatment on diagnosis is desirable. Bronchial artery embolization (BAE) is the first choice for treatment because of its minimally invasive nature.

**Case presentation:**

A 74-year-old man was referred to our department and pointed out a tumor-like lesion under the aortic arch on a contrast-enhanced computed tomography (CECT). We diagnosed it as a mediastinal BAA, and BAE was planned. The bronchial artery arising from the aorta showed strong atherosclerotic meanders and was difficult to engage, resulting in iatrogenic acute aortic dissection (AAD). Urgent thoracic endovascular aortic repair (TEVAR) was performed. There was no contrast effect of the aneurysm on a postoperative CECT, indicating thrombotic occlusion.

**Conclusions:**

We were preparing a system that could convert to surgery or TEVAR, in case of difficult to treat with BAE or ruptured aneurysm during BAE, so we were able to respond quickly to aortic dissection caused by BAE and obtain a good result of both BAA and aortic dissection. TEVAR would be considered a treatment of BAA, in cases such as the present case, where engagement is difficult, where the length of the inflow artery is short, or where the inflow artery is tortuous.

## Background

BAA is classified into the mediastinal type and intrapulmonary type according to the site of occurrences and found in < 1% of patients undergoing bronchial arteriography [1–4]. Although surgical and endovascular treatment are the two recommended treatments for BAA [1, 5], BAE could be the first choice for asymptomatic mediastinal BAA [3]. We report a case of iatrogenic AAD dissection during BAE for mediastinal BAA, in which TEAVR was performed with good results for both BAA and iatrogenic AAD, and we propose a treatment strategy for mediastinal BAA based on previous reports.

## Case presentation

**Fig. 1:**
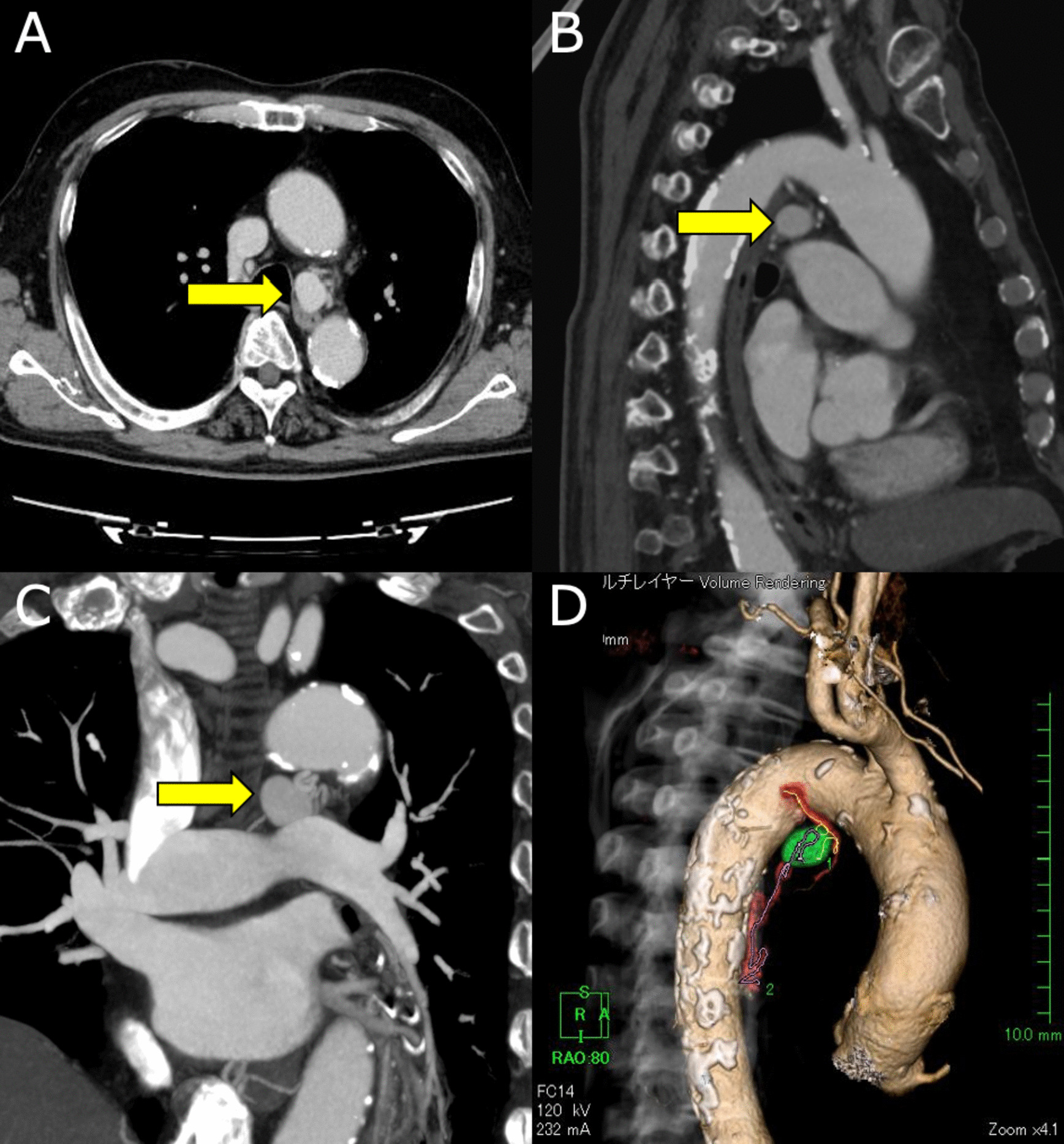
CECT images showing an aneurysm originating from the dorsal mediastinal side of the distal arch. **A** Axial view. **B** Sagittal view. **C** Coronal view. Yellow arrows in **A**, **B**, and **C** indicate the aneurysm. **D** Three-dimension CT shows an aneurysm (green) and an inflow artery (red)

A 74-year-old man who was referred to our department pointed out the tumor-like lesion under the aortic arch on CECT. CECT showed an aneurysm originating from the dorsal mediastinal side of the distal arch (Fig. 1). It was 21 × 17 × 17 mm in size, and the inflow artery diameter was 2.5 mm. We diagnosed it as a mediastinal-type BAA, and BAE was planned. He had a history of bronchiectasis, soft tissue tumor, hypertension, and diabetes mellitus.

**Fig. 2:**
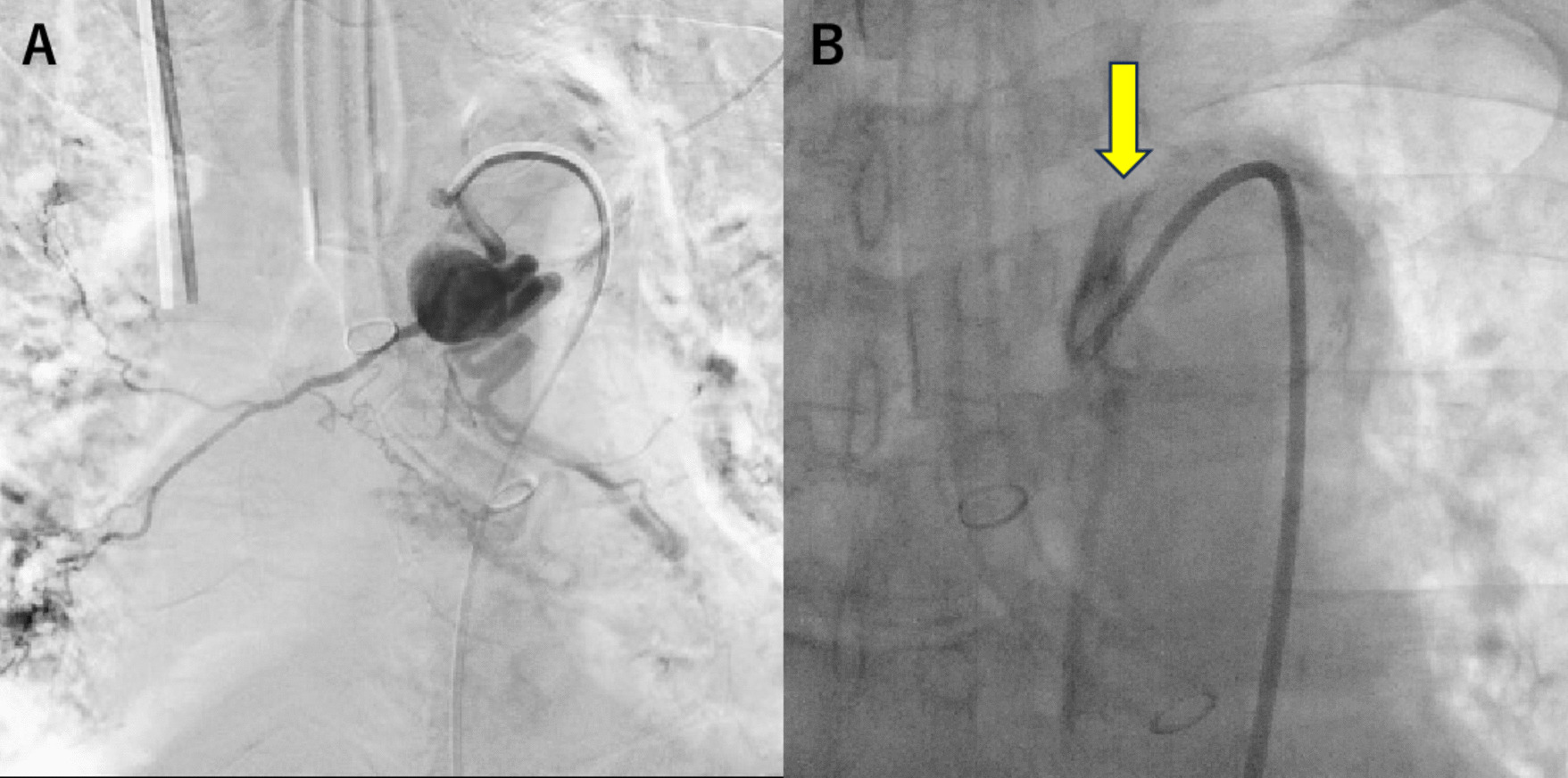
Bronchial angiography. **A** The aneurysm was contrasted. **B** Yellow arrow indicating contrast residue along the aortic wall

**Fig. 3:**
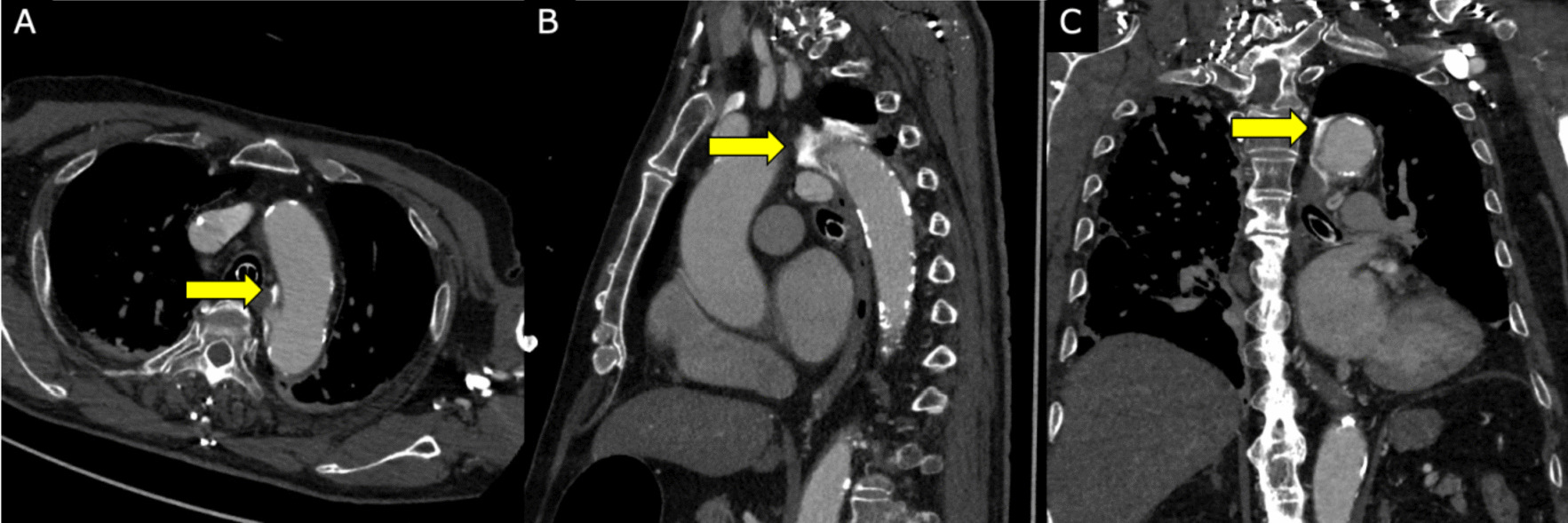
CECT image of iatrogenic acute aortic dissection. It shows a localized dissection with a 1–5-mm false lumen opening from the bronchial artery origin to the distal arch. Yellow arrows indicate contrast leakage. **A** Axial view. **B** Sagittal view. **C** Coronal view

**Fig. 4:**
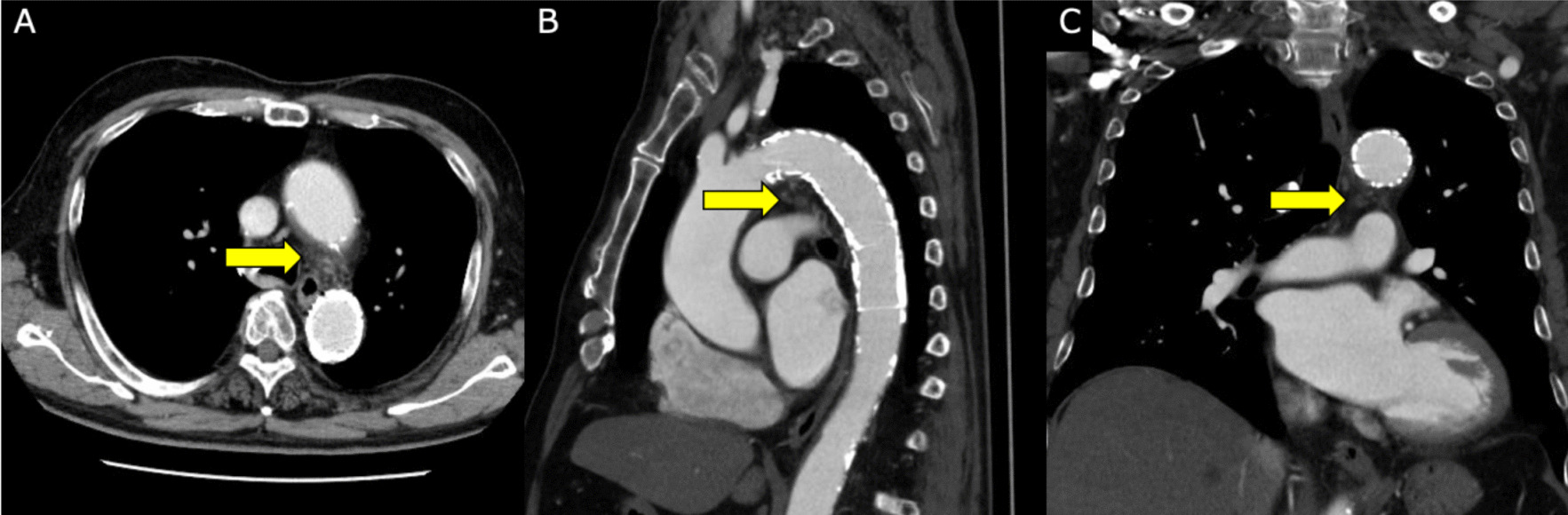
CECT image of 6 months after TEVAR. Yellow arrows indicate the aneurysm with no contrast effect, and its diameter is reduced. **A** Axial view. **B** Sagittal view. **C** coronal view

Under general anesthesia in case it ruptured during treatment, the following two preparations were made: to be able to shift to TEVAR, cut down the right femoral artery and place a 5-Fr sheath and to be able to shift to surgical hemostasis, disinfect, and drape the chest to groin area for a median sternotomy. Although the aneurysm was contrasted in bronchial arteriography, the bronchial artery was difficult to engage due to strong atherosclerotic tortuosity. A 4-Fr guide catheter (Medikit, Tokyo, Japan) was advanced to the origin of bronchial artery; the aneurysm was contrasted (Fig. 2A). Even though it could be contrasted, the bronchial artery was difficult to engage due to strong atherosclerotic tortuosity and its location of branching from distal arch. We replaced the 7-Fr sheath and changed to 7-Fr guide catheter (Boston Scientific, Tokyo, Japan) with better stability. After the guide catheter managed to engage the origin of the bronchial artery, a micro catheter was advanced through the guide catheter. While advancing the micro catheter, the guide catheter was slipped out of place, and the bronchial artery no longer contrasted. In addition to that contrast, residue along the aortic wall was observed (Fig. 2B). We noticed that a localized aortic dissection occurred in the aortic arch near the beginning of the bronchial artery. BAE was discontinued, and CECT evaluation was performed. A localized dissection with 1–5-mm false lumen opening was observed from the bronchial artery origin to the distal arch (Fig. 3). Urgent TEVAR was performed from the peripheral of the left subclavian artery to Th7 level using GORE®TAG®Conformable Thoracic Stent Graft. The operation time was 84 min, and the blood loss was 10 mL. The patient’s postoperative course was uneventful, and CECT at postoperative day 7 showed no contrast effect of the BAA or no endoleak. He was discharged home 9 days after surgery. CECT on 6 months postoperatively showed no contrast effect on the BAA, the aneurysm diameter was reduced to 5.6 mm, and endoleak was not observed (Fig. 4).

## Discussion and conclusions

BAA is a very rare disease found in < 1% of patients undergoing bronchial arteriography [1, 2]. It is classified into the mediastinal type and intrapulmonary type according to the site of occurrence, with the former, being reported even less frequently [3, 4]. The mediastinal type is often asymptomatic, and rupture may cause aortic dissection-like symptoms, chest and back pain, and circulatory failure [6, 7]. On the other hand, the intrapulmonary type is detected with hemoptysis or bloody sputum as the main complaint [8]. BAA is fatal if ruptured, so it should be treated regardless of aneurysm size even if it is asymptomatic [5]. The present case was also asymptomatic but was discovered incidentally during a close examination for another disease. With the recent development of diagnostic imaging, the number of reports of asymptomatic cases is increasing [9]. Regarding the etiology of bronchial artery aneurysms, Sato et al. reported that there is a condition of increased blood flow in bronchial arteries; then, inflammation is added to this condition to form an aneurysm in the weakened part of the arterial wall [10], suggesting that multiple factors may be involved about concerning causation. The patient had a history of bronchiectasis, which was considered to be a contributing factor.

Surgical or endovascular treatment is recommended for BAA, and the latter could be the first choice for asymptomatic mediastinal BAA [1, 3]. Surgical procedures for mediastinal BAA include resection of the aneurysm or ligation of the inflow artery through thoracotomy [1, 6, 11], which are highly curative but also highly invasive. Thus, it is difficult to perform surgery in patients with poor pulmonary function or poor general condition [1, 12]. Endovascular treatment for mediastinal BAA includes BAE or TEVAR, which are less invasive compared to surgery [1, 6, 9, 11, 12]. However, TEVAR is not currently covered by insurance for BAA in Japan, so it is a difficult choice for some. The problem with BAE is revascularization of the aneurysm, which can be caused by collateral vessels, incomplete embolization, and arterial recanalization [12]. Also, the BAA itself makes BAE difficult under the following conditions, where the inflow artery is short neck, tortuous, thin, or multiple [1] and where the engagement is difficult because of acute angle or location of inflow artery bifurcation [12].

**Fig. 5:**
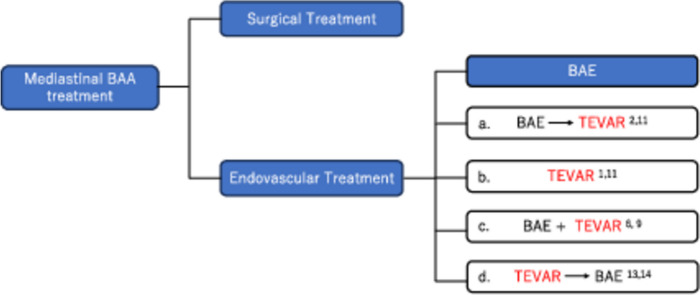
Mediastinal BAA treatment. White squares (**a–d**) show no insurance coverage in Japan; thus, it is difficult to choose. However, if it is a difficult case of BAE such as where the inflow artery is short neck, tortuous, thin, or multiple and where the engagement is difficult because of acute angle or location of inflow artery bifurcation, it would be better to choose if possible

Although it is difficult to select TEVAR, there have been useful reports of mediastinal BAA treatment with TEVAR in clinical practice [1, 2, 6, 9, 11, 12]. In previously reported cases where TEVAR has been performed for mediastinal BAA are summarized in Fig. 5. There were two cases (Fig. 5a) of BAE converted to TEVAR: a case of ruptured aneurysm treated with BAE, which was converted to TEVAR after unsuccessful treatment [2], and a case of recanalization 9 days after BAE and additional treatment with TEVAR [12]. There were two cases (Fig. 5b): TEVAR performed from the beginning due to difficulty of BAE [1, 11]. There were two cases (Fig. 5c): BAE and TEVAR were combined from the beginning to prevent recanalization and coil deviation after embolization in case of short neck [6, 9]. As for the cases in which TEVAR was performed after BAE, they were originally considered difficult cases of BAE, and if TEVAR is covered by insurance in the future, they may be able to choose clinically appropriate treatments. There were also two cases (Fig. 5d) in which BAE was performed for endoleak after TEVAR [13, 14], whereas there were some cases of successful treatment with TEVAR alone including our case [1, 11]. The criteria for whether TEVAR alone is sufficient to complete the treatment of mediastinal BAA will require further case accumulation. Clinically, TEVAR could be a treatment option for mediastinal BAA.

It was fortunate that this case did not extend to retrograde type A aortic dissection, but we should have opted for surgery or TEVAR instead of BAE from the beginning, since it was a difficult case of BAE. We were preparing a system that could convert to surgery or TEVAR, in case of difficult to treat with BAE or ruptured aneurysm during BAE, so we were able to respond quickly to aortic dissection caused by BAE and obtain the good result of both BAA and aortic dissection.

## Data Availability

All data supporting the conclusions of this article are included within the published article.

## Abbreviations

CECT: Contrast-enhanced computed tomography
BAA: Bronchial artery aneurysm
BAE: Bronchial artery embolization
TEVAR: Thoracic endovascular aortic repair
